# Missed opportunities in nutritional care: prevalence, mortality, and resource utilization in internal medicine wards

**DOI:** 10.3389/fnut.2026.1755750

**Published:** 2026-05-13

**Authors:** Ricardo C. Marinho, Ana Craveiro, Susana Ferreira, Elisabete Carolino, Marta S. Lopes, João A. Correia, Anibal Marinho, Marisa D. Santos

**Affiliations:** 1 Clinica de Anestesiologia, Medicina Intensiva, Emergência e Urgência, Centro Hospitalar Universitário de Santo António, Unidade Local de Saúde de Santo António, Porto, Portugal; 2 School of Medicine and Biomedical Sciences (ICBAS), University of Porto, Porto, Portugal; 3 Direção de Planeamento e Controlo de Gestão, Centro Hospitalar Universitário de Santo António, Unidade Local de Saúde de Santo António, Porto, Portugal; 4 H&TRC – Health and Technology Research Center, ESSL – Escola Superior de Saúde de Lisboa, Instituto Politécnico de Lisboa, Lisbon, Portugal; 5 Hematology Unit, Unidade Local de Saúde Entre Douro e Vouga, Santa Maria da Feira, Portugal; 6 Departamento de Ciências Médicas, Universidade de Aveiro, Aveiro, Portugal; 7 Clinica de Medicina, Centro Hospitalar Universitário de Santo António, Unidade Local de Saúde de Santo António, Porto, Portugal; 8 Colorretal Surgery Unit, Clinica de Cirurgia, Centro Hospitalar Universitário de Santo António, Unidade Local de Saúde de Santo António, Porto, Portugal; 9 Unit for Multidisciplinary Research in Biomedicine (UMIB), School of Medicine and Biomedical Sciences (ICBAS), University of Porto, Porto, Portugal; 10 ITR - Laboratory for Integrative and Translational Research in Population Health, Porto, Portugal

**Keywords:** health costs and outcomes, hospital mortality, internal medicine, malnutrition, nutritional risk, health economics, real-world evidence, NRS-2002

## Abstract

**Background:**

Malnutrition is a highly prevalent and underdiagnosed condition among hospitalized patients, especially in internal medicine wards. Hospital malnutrition is associated with increased morbidity, mortality, prolonged admissions, and a substantial economic burden.

**Objectives:**

This study aimed to assess the clinical and economic impact of nutritional risk in patients admitted to internal medicine wards, focusing on mortality, hospital resource utilization, and the effectiveness of nutritional interventions.

**Methods:**

A retrospective cohort study was conducted at ULS Santo António, Porto, including 1,150 hospital admissions from January to December 2022. All adult patients with nutritional risk screening (NRS-2002) in the first 48 h of admission were included. Data were collected from hospital information systems on demographic, clinical, and economic variables, with outcomes including in-hospital mortality, readmissions at 30, 90, and 180 days, and one-year post-discharge mortality.

**Results:**

Nutritional risk (NRS-2002 ≥ 3) was identified in 42.4% of patients (*n* = 488), while ICD-10 malnutrition coding at discharge was recorded in only 0.7% of admissions. Of patients at nutritional risk, 74.4% (*n* = 363) received no nutritional supplementation. Nutritional risk was associated with higher in-hospital mortality, longer length of stay, and increased costs across all resource categories. In the time-dependent Cox model, patients at nutritional risk without supplementation showed a markedly higher hazard of in-hospital death at admission (HR 23.32, 95% CI 13.09–41.56), with this excess hazard attenuating over time. Patients at nutritional risk who received supplementation also showed elevated early risk (HR 6.15, 95% CI 2.96–12.80), though lower than unsupplemented patients. A similar pattern was observed for one-year post-discharge mortality. Total hospitalization costs were approximately 79% higher in at-risk patients, driven mainly by longer length of stay.

**Conclusion:**

Nutritional risk affected 42.4% of internal medicine inpatients and was associated with higher mortality and resource use. The finding that 74.4% of at-risk patients received no nutritional intervention represents a substantial missed opportunity. Patients who received nutritional supplementation showed a pattern of lower mortality risk than unsupplemented at-risk patients, consistent with but not proving a beneficial association. These findings support systematic nutritional screening and timely intervention in hospital care.

## 1 Introduction

Malnutrition is a highly prevalent and under-recognized condition among hospitalized patients, particularly those admitted to internal medicine wards. Recent multicenter studies have reported that the prevalence of malnutrition or nutritional risk in these settings ranges from 48% to over 73%, depending on the assessment tools and populations studied (1, 2). These high rates are consistent across diverse healthcare systems and underscore the critical need for systematic nutritional assessment in hospitalized adults.

The clinical consequences of malnutrition in the hospital setting are profound. Patients identified as malnourished or at nutritional risk experience significantly higher rates of morbidity and mortality. Large cohort studies and meta-analyses have demonstrated that malnutrition is independently associated with increased odds of in-hospital mortality (3–5). In addition, malnutrition is linked to longer hospital stays, higher rates of complications, and increased likelihood of readmission, all of which contribute to greater healthcare utilization (6).

The economic burden of hospital malnutrition is substantial. Cost-of-illness models estimate that malnutrition imposes billions of dollars in additional healthcare costs annually, driven primarily by prolonged length of stay and increased resource use (7–9).

Early identification of patients at nutritional risk is essential to mitigate these adverse outcomes. The Nutritional Risk Screening 2002 (NRS-2002) is a validated tool with high sensitivity and specificity for detecting nutritional risk in hospitalized adults (10, 11). Its use has been recommended for routine screening upon hospital admission and during the hospital stay to enable timely intervention and prevent further nutritional decline.

Importantly, randomized controlled trials and meta-analyses have shown that individualized nutritional support for at-risk inpatients reduces short-term mortality and hospital readmission (12). Despite this evidence, a substantial proportion of patients identified as at nutritional risk do not receive appropriate nutritional intervention, highlighting a persistent care gap in clinical practice (13).

While multiple studies have documented the prevalence of nutritional risk and its association with clinical outcomes, few have integrated patient-level cost data using a real-patient costing methodology within the same cohort. This study contributes novel Real World Evidence by simultaneously examining mortality, readmissions, and granular economic impact, including the cost of the implementation gap, within a single well-characterized internal medicine cohort, using validated screening tools and an accurate cost-attribution framework.

This study aims to evaluate the clinical and economic impacts of nutritional risk among patients admitted to internal medicine departments, focusing on mortality, hospital resource utilization, and the association of nutritional interventions with observed outcomes, in an acute hospital setting. By clarifying the relationship between nutritional status and health outcomes, this research seeks to inform evidence-based strategies to improve patient care and optimize resource allocation in hospital settings.

## 2 Materials and methods

### 2.1 Study design and population

This retrospective observational cohort study was conducted at the Internal Medicine Department of ULS Santo António, Porto, Portugal, from January to December 2022. The study followed the *Strengthening the Reporting of Observational Studies in Epidemiology* (STROBE) guidelines for observational research.

The hospital is a tertiary-level academic medical center that serves as a reference hospital for the northern region of Portugal, with approximately 1,200 beds and an Internal Medicine department comprising multiple wards with a total capacity of 120 beds.

### 2.2 Inclusion and exclusion criteria

The study included adult inpatients (≥18 years) admitted to Internal Medicine wards with a minimum hospital stay of 24 h and complete nutritional risk screening performed within 48 h of admission. Since all information was collected from hospital electronic registries, all inpatients had complete demographic and clinical records. Inpatients receiving terminal or palliative care were excluded from the analysis.

### 2.3 Sample size and recruitment

All inpatient admissions with a Nutritional Risk Screening 2002 (NRS-2002) registry in *S-Clinico* (Electronic Clinical Registry) in 2022 were collected, totaling 1,564 inpatient admissions. For study purposes, all inpatient admissions with incomplete NRS-2002 screening registries were excluded, as were all admissions not in internal medicine wards. The final study population comprised 1,150 inpatient admissions, meeting the inclusion criteria (Supplementary Figure S1). No formal sample size calculation was performed, as this was a comprehensive analysis of all eligible patients during the study period.

The requirement for NRS-2002 screening within 48 h of admission introduced a structural selection bias. Patients admitted during weekends (Friday–Sunday) or directly from the Emergency Department are less likely to undergo timely screening within the prescribed window. Similarly, terminally ill patients are not routinely screened in this institution. These groups are likely to carry a higher burden of nutritional risk and illness severity. Their systematic exclusion may lead to an underestimation of nutritional risk prevalence and a survival advantage in the analytic cohort compared to the true hospital population. A comparison between included and excluded admissions was not possible with the available data, as non-screened patients lacked complete demographic and clinical records.

### 2.4 Nutritional risk screening protocol

Nutritional risk assessment was performed using the Nutritional Risk Screening 2002 (NRS-2002) tool (10), as recommended by ESPEN for hospital settings (14). The NRS-2002 was selected for its validated predictive validity for clinical outcomes and its widespread implementation in Portuguese hospitals, following national healthcare system mandates. The screening was conducted by trained nursing staff within 48 h of patient admission. Nurses underwent a formal training program specifically designed to standardize the application of the NRS-2002 tool, emphasizing the importance of early nutritional risk detection and correct data entry. Additionally, monthly audits were carried out to evaluate compliance with the screening protocol, identify potential gaps, and provide feedback to maintain high-quality screening practices across the hospital wards.

The NRS-2002 assessment consisted of two sequential phases. The initial screening phase included four screening questions administered within 48 h of admission: assessment of BMI < 20.5 kg/m^2^, weight loss within the last 3 months, reduced dietary intake in the last week, and severe illness status. For patients with positive initial screening (≥1 affirmative answer), a detailed assessment was performed including nutritional status scoring (0–3 points) based on BMI, percentage weight loss, and food intake reduction; disease severity scoring (0–3 points) for stress metabolism assessment based on underlying medical condition; and age adjustment with one additional point for patients ≥70 years.

Patients with a total score ≥3 were classified as having nutritional risk present and requiring nutritional intervention, while those with a score <3 underwent weekly re-screening during hospitalization. The NRS-2002 screening was integrated into the hospital’s *SClínico* electronic health record system, the standardized platform used across Portuguese National Health System hospitals, featuring automatic screening prompts upon patient admission, structured data entry with mandatory completion fields, real-time risk score calculation and classification, and direct communication pathways to nutrition teams for positive screens.

### 2.5 Data collection and management

Comprehensive data collection utilized multiple integrated hospital information systems including *SClínico* electronic health record demographics, clinical data, and screening results; Hospital Information System (SONHO) for admission/discharge data and length of stay. Morbidity data (ICD-10 diagnoses and procedural coding) were obtained from the National NHS morbidity database (SIMH). In this study, case complexity was summarized using the Diagnosis Related Group (DRG) relative weight assigned to each inpatient admission. Relative weight is a unitless index that reflects the expected resource use and clinical complexity of the DRG compared with an average reference case (relative weight = 1.0); values greater than 1.0 indicate admissions that are expected to consume more resources and represent higher clinical complexity, whereas values below 1.0 indicate less complex, less resource-intensive cases.

Demographic and Clinical Characteristics included age, sex, admission and discharge dates, primary and secondary diagnoses using ICD-10 coding, and Charlson Comorbidity Index calculation using validated ICD-10-based methodology. The Charlson Comorbidity Index was calculated from ICD-10 coding in discharge notes using an Excel-based ICD-10 dataset calculator (15). ICD-10 malnutrition coding was assessed using codes E 43, E 440 and E441.

Nutritional Assessment Data encompassed complete NRS-2002 screening results and individual component scores, anthropometric measurements including BMI and weight loss percentage. For admission costing purposes, medication and nutritional supplement costs attached to each admission collected from Pharmacy Management System (GHAF—*Gestão Hospitalar de Armazéns e Farmácia*), the institutional pharmacy dispensing and stock management system, at purchasing price; for inpatients days at ward costing, analytic accounting data used, for diagnostic and therapeutic procedures attached to each inpatient admission, legally fixed price used. In this study, nutritional support was identified indirectly through admission-level expenditure on nutritional products retrieved from the GHAF. An important limitation of this approach should be noted at the outset: the GHAF system aggregates all pharmaceutical nutritional products—oral supplements, enteral preparations, and parenteral solutions—under a single cost category. As a result, no information on dosage, duration, or modality of administration is available, nor do they capture dietetic counselling without product prescription. This exposure misclassification is acknowledged and may attenuate or distort the observed associations between supplementation and clinical outcomes. For the purposes of the present analyses, any non-zero cost in this category during the index admission was classified as “nutritional supplementation,” whereas admissions with zero expenditure were considered as having received no recorded nutritional supplementation. All comparative statements between supplemented and non-supplemented groups should be interpreted with this limitation in mind.

Primary outcomes were in-hospital mortality and length of hospital stay. Secondary outcomes included 30, 90, and 180-day hospital readmissions, and 1-year post-discharge mortality. Economic outcomes comprised comprehensive healthcare resource utilization and costs. For readmission analyses, patients who died during the index admission were excluded from the denominator, as they had no opportunity for readmission. Post-discharge survival analyses were similarly left-truncated at the date of hospital discharge.

### 2.6 Cost analysis framework

Cost analysis encompassed all cost factors. A real patient costing approach used. For that purpose, all direct costs from detailed medicines and drugs, detailed nutritional products, diagnostic and specialized procedures attached to each case are monetized and not the average cost. For indirect costs, ward costing is calculated by the average daily cost for each specific ward, accounting for all human and technical resources costs, following the complete inpatient course, since not every medical ward has the same resource allocation. General infrastructural hospital costs are allocated based on ward-day consumption. Total inpatient costs result from the comprehensive summation of all cost categories. The cost structure analysis revealed that daily hospitalization costs were the largest contributor to total costs, followed by medication costs.

### 2.7 Statistical analysis

The data were analyzed using statistical software SPSS, version 29.0 for Windows. Results were considered significant at a 5% significance level. A data quality analysis was performed on the generated database, including an analysis of missing values. All variables under study contained information for all patients. The Kolmogorov–Smirnov test was used to assess the normality of the data. For sample characterization, frequency analysis (n, %) was used for qualitative data, and descriptive measures including minimum, maximum, mean, and standard deviation were used for quantitative data. To identify cost predictors, linear regression analysis was employed using the Stepwise selection method for the variables to be included in the models. Since the data did not meet the normality assumption, Box-Cox transformations were applied to normalize the data. The models obtained adhere to the Gauss-Markov conditions (residuals with zero mean, constant variance, and normal distribution) and confirm the absence of multicollinearity. The regressors considered for costs included age, sex, length of stay, disease severity, NRS-2002 and Charlson Comorbidity Index. Sensitivity analyses were conducted by comparing models obtained using different variable selection procedures (backward, forward, and LASSO), varying selection criteria, and fitting a full model including all candidate covariates. Bootstrap resampling was used to assess model stability. To evaluate the influence of nutritional risk and comorbidities on mortality, Survival Analysis was conducted using Cox Regression, employing the Forward Stepwise (Conditional LR) method for variable selection in the model. The proportional hazards assumptions were tested using Schoenfeld residual analysis and visual inspection of partial residual plots. To study the relationship between nutritional risk and expenditure on nutritional supplements, the relationship between mortality and disease severity with costs for nutritional supplements, the Chi-Square test (when the assumptions were verified), Fisher’s exact test, Chi-Square test by Monte Carlo simulation and t test were used (because the size of each group was large, although the assumption of normality was not verified and even present very high standard deviations in some situations. However, the results were always confirmed using the Mann–Whitney test). Measures of effect size in the comparison of groups and their evaluation were also presented in accordance with the recommendation of the American Statistical Association. To identify regressors of nutritional risk, Binary Logistic Regression was used, as well as to identify regressors of readmissions. To study the relationship between comorbidities and nutritional risk with mortality, survival analysis was used, using Cox Regression.

### 2.8 Ethical considerations

The study protocol was approved by the institutional ethics committee of ULS Santo António. Given the retrospective nature of the study, which used routinely collected clinical data, informed consent was waived. All data were de-identified and handled in accordance with the European Union General Data Protection Regulation (GDPR). Patient confidentiality was maintained throughout the study period, and all analyses were conducted on anonymized datasets.

## 3 Results

The study cohort comprised of 1,150 patients hospitalized in Internal Medicine ward from January to December 2022, with ages ranging from 18 to 106 years, and an average age of 71.79 ± 17.28 years and a median age of 75 years. Most patients were aged 80 or older (*n* = 443, 43%) and female (*n* = 656, 57%). The nutritional risk as assessed by the NRS-2002 was present in 42.4% (*n* = 488) of patients; however, at discharge, the ICD 10 coding was only 0.7% (Table 1). The overall in-hospital mortality rate was 17.1% (*n* = 197) (Supplementary Table S1). One year after discharge, the overall mortality rate was 11.4% (*n* = 131).

**Table 1:** Characteristics of the studied participants (*n* = 1,150).

Sample characteristics	Total (*n* = 1,150)	NRS < 3 (*n* = 662)	NRS ≥ 3 (*n* = 488)	*p*-value	Effect size
Male sex, n (%)	494 (43)	275 (41.5)	177 (24.3)	**0.259** ^**1**^	0.033^3^
Age (years), mean ± SD	71.79 ± 17.28	67.67 ± 18.31	77.39 ± 13.98	**<0.001** ^2*^	**0.695** ^**4**^
Relative Weight, mean ± SD	1.01 ± 1.81	1.1 ± 1.5	0.89 ± 2.09	**0.070** ^**2**^	**0.100** ^**4**^
Charlson comorbidity index, mean ± SD	5.18 ± 3.32	4.63 ± 3.34	5.93 ± 3.14	**0.001** ^2*^	**0.115** ^**5**^
Length of stay (days), mean ± SD	18.90 ± 31.07	14.60 ± 24.61	24.73 ± 37.36	**<0.001** ^2*^	**0.271** ^**5**^
Hospital Mortality, n (%)	197 (17.1)	55 (8.3)	142 (29.1)	**<0.001** ^1*^	**0.273** ^**3**^
Mortality at 1 year after discharge	131 (11.4)	72 (10.9)	59 (12.1)	0.522^1^	0.019^3^
Readmissions up to 30 days, n(%)	89 (7.7)	57 (8.6)	32 (6.6)	0.198^1^	0.038^3^
Readmissions up to 90 days, n(%)	127 (11.0)	83 (12.5)	44 (9.0)	0.060^1^	0.056^3^
Readmissions up to 180 days, n(%)	157 (13.7)	105 (15.9)	52 (10.7)	0.011^1*^	0.075^3^
Total costs, mean ± SD	10446.75 ± 20182.94	7820.45 ± 391.80	28302.91 ± 1281.21	<0.001^2*^	0.219^**4**^
Medication costs, mean ± SD	1590.68 ± 6886.40	1156.23 ± 3693.70	2180.04 ± 9631.37	0.026^2*^	0.106^4^
Nutritional Support costs, mean ± SD	14.76 ± 100.19	2.72 ± 13.79	31.09 ± 151.52	<0.001^2*^	0.187^4^
Antibacterial costs, mean ± SD	186.68 ± 1125.60	99.59 ± 498.52	304.83 ± 1,620	<0.001^2*^	0.127^4^
Costs for complementary diagnostic means, mean ± SD	774.50 ± 1115.93	702.19 ± 834.28	872.6 ± 1405.93	**0.017** ^2*^	**0.238** ^**4**^
Daily hospitalization costs, mean ± SD	8081.56 ± 16362.70	5962.03 ± 8089.97	10958.84 ± 22988.33	<0.001^2*^	0.335^4^
Comorbidities (ICD-10)					
AIDS, n (%)	12 (1)	9 (1.4)	3 (0.6)	**0.255** ^**1**^	0.036^3^
Solid tumour with metastasis, n (%)	101 (8.8)	46 (6.9)	55 (11.3)	**0.010** ^1*^	0.075^3^
Any tumour without metastasis, n (%)	162 (14.1)	83 (12.5)	79 (16.2)	**0.079** ^**1**^	0.052^3^
Moderate or severe liver disease, n (%)	44 (3.8)	26 (3.9)	18 (3.7)	**0.835** ^**1**^	**0.006** ^**3**^
Mild liver disease, n (%)	27 (2.3)	18 (2.7)	9 (1.8)	**0.333** ^**1**^	0.029^3^
Diabetes with end organ damage, n (%)	111 (9.7)	59 (8.9)	52 (10.7)	**0.322** ^**1**^	0.029^3^
Diabetes without end organ damage, n (%)	184 (16)	108 (16.3)	76 (15.6)	**0.735** ^**1**^	**0.010** ^**3**^
Moderate or severe kidney disease, n (%)	169 (14.7)	83 (12.5)	86 (17.6)	**0.016** ^1*^	**0.071** ^**3**^
Peptic ulcer, n (%)	7 (0.6)	3 (0.5)	4 (0.8)	**0.430** ^**1**^	**0.023** ^**3**^
Hemiplegia, n (%)	28 (2.4)	14 (2.1)	14 (2.9)	**0.412** ^**1**^	**0.024** ^**3**^
Dementia, n (%)	174 (15.1)	69 (10.4)	105 (21.5)	**<0.001** ^1*^	**0.153** ^**3**^
Cerebrovascular disease, n (%)	64 (5.6)	35 (5.3)	29 (5.9)	**0.632** ^**1**^	**0.014** ^**3**^
Heart failure, n (%)	325 (28.3)	183 (27.6)	142 (29.1)	**0.588** ^**1**^	**0.016** ^**3**^
Connective tissue disease, n (%)	53 (4.6)	38 (5.7)	15 (3.1)	**0.033** ^1*^	**0.063** ^**3**^
Chronic obstructive pulmonary disease, n (%)	168 (14.1)	101 (15.3)	67 (13.7)	**0.469** ^**1**^	**0.021** ^**3**^
Peripheral vascular disease, n (%)	57 (5)	31 (4.7)	26 (5.3)	**0.618** ^**1**^	**0.015** ^**3**^
Acute coronary syndrome, n (%)	48 (4.2)	23 (3.5)	25 (5.1)	**0.167** ^**1**^	**0.041** ^**3**^

AIDS, acquired immunodeficiency syndrome; NRS-2002, nutritional risk screening 2002; SD, standard deviation. *Statistically significant differences at the 5% significance level. ^1^Chi-square test; ^2^Two independent samples *t*-test, ^3^Cramer’s V, ^4^Glass’s delta, ^5^Cohen’s d.

Comparison between nutritional risk groups revealed homogeneity in sex distribution (*p* = 0.259, effect size = 0.033, negligible effect), but significant age differences (*p* < 0.001, effect size = 0.695, large effect), with patients at nutritional risk being substantially older (Table 1).

Regarding patient complexity, on average, patients had a relative weight of 1.01 ± 1.81 (range: 0–25.32), with 674 (58.6%) presenting a lower level of complexity. Concerning the Charlson comorbidity index, patients presented an average index of 5.18 ± 3.32 (range 0–18), indicating moderate to high comorbidity burden across the cohort. Between-group comparisons showed no significant differences in complexity levels (*p* = 0.070, effect size = 0.100, small effect), but patients at nutritional risk had higher Charlson Comorbidity Index scores (*p* = 0.001, effect size = 0.115, small effect).

Regarding comorbidities, those with the highest prevalence rate were congestive heart failure (*n* = 325, 28.3%), diabetes mellitus without complications (*n* = 184, 16.0%), dementia (*n* = 174, 15.1%), chronic kidney disease (*n* = 169, 14.7%), chronic pulmonary disease and pneumonia (*n* = 168, 14.6%), cancer without metastases (*n* = 162, 14.1%), diabetes mellitus with complications (*n* = 111, 9.7%) and cancer with metastases (*n* = 101, 8.8%).

Specific comorbidities including solid tumour with metastasis, moderate or severe renal disease, and dementia showed statistically significant but clinically minimal differences between groups (*p* < 0.05, effect sizes <0.2, small effects) (Table 1).

The hospital length of stay varied considerably, ranging from 1 to 436 days with a mean of 18.90 ± 31.07 days. Patients at nutritional risk had significantly longer stays compared to those without risk (*p* < 0.001, effect size = 0.273, small to medium effect). Regarding mortality, significant differences were observed between nutritional risk groups (*p* < 0.001, effect size = 0.273, small to medium effect), with higher mortality rates among patients at nutritional risk. Readmission patterns showed temporal variation: higher rates at 90 and 180 days compared to 30 days across both groups. Between-group comparisons revealed no significant differences in readmissions at 30 and 90 days (*p* > 0.05, very small effect sizes), but patients at nutritional risk showed higher readmission rates at 180 days (*p* = 0.011, effect size = 0.075, very small effect) (Table 1).

As predictors of nutritional risk, age, sex, and Charlson comorbidity index were identified. It was found that for each additional year of age, the risk of malnutrition increases by 3.6% [Odds ratio = 1.036, 95% C. I. = (1.027; 1.046)] and for each additional unit in the Charlson comorbidity index the risk of malnutrition increases by 5.6% [Odds ratio = 1.056. 95% C. I = (1.014; 1.100)]. Women have a risk of malnutrition approximately 29% lower than men [Odds ratio = 0.711, 95% C. I = (0.552; 0.917)] (Table 2).

**Table 2:** Identification of nutritional risk predictors.

Number of steps	Variables in the equation	*B*	S. E.	Wald	df	*P*	Odds ratio	95% C. I. for EXP(B)	
								Lower	Upper
Step 3	Age	0.036	0.005	59.278	1	<0.001	1.036	1.027	1.046
	Sex (female)	−0.340	0.130	6.896	1	0.009	0.711	0.552	0.917
	Charlson comorbidity index	0.055	0.021	6.970	1	0.008	1.056	1.014	1.100
	Constant	−3.019	0.325	86.504	1	<0.001	0.049		

Variable(s) entered on step 1: Age. Variable(s) entered on step 2: Charlson Comorbidity Index. Variable(s) entered on step 3: Sex. Results of Binary Logistic Regression, with Forward Conditional method for selecting variables to be included in the model.

Comprehensive cost analysis revealed significant differences between nutritional risk groups across all cost categories (*p* < 0.05), with small to medium effect sizes. The categories were total costs, with medication, with nutritional support, with antibacterial drugs, with complementary diagnostic means and with daily hospitalization. Being at nutritional risk increases daily hospitalization costs by approximately 83%, medication costs by 88.5%, antibacterial costs by 206%, diagnostic and specific procedure costs by 24%, and total costs by 79% (Table 1).

Regarding total costs, the predictors identified were length of stay (the strongest predictor—greater Beta value), nutritional risk, and age. It was found that longer length of stay and risk of malnutrition are associated with higher costs, while older age is associated with lower costs. Sensitivity analyses were conducted using bootstrap resampling (1,000 replications, BCa method) and alternative variable selection procedures (forward and backward stepwise). The results were highly consistent across approaches, with length of stay, NRS-2002 classification, and age remaining significant predictors. Bootstrap estimates and confidence intervals were similar to the original model, indicating robustness of the findings (Table 3). It should be noted that total hospitalization costs are inherently co-determined by length of stay; therefore, the association between nutritional risk and total costs likely operates substantially through its effect on prolonging hospitalization. The independent contribution of NRS score to costs, beyond its effect through length of stay, should be interpreted cautiously given the collinearity between these variables in the regression models.

**Table 3:** Identification of total costs predictors.

Number of models	Variables in the model		Unstandardized Coefficients			Standardized Coefficients	*t*	*p*	95.0% Confidence interval for B		Collinearity statistics
			B	Bias	Std. error	Beta			Lower bound	Upper bound	VIF
3	(Constant)	Stepwise	7849.206	−59.386	1998.833		3.927	<0.001	3927.421	11770.991	
		Bootstrap	7908.592		2525.813			0.008	3494.095	11447.765	
	Length of stay	Stepwise	395.625	−0.640	15.355	0.601	25.764	<0.001	365.497	425.753	1.026
		Bootstrap	396.265		82.077			0.001	286.153	657.324	
	NRS-2002 Classification	Stepwise	4411.834	−87.538	986.495	0.109	4.472	<0.001	2476.293	6347.374	1.112
		Bootstrap	4324.296		1216.652			0.002	2198.082	6079.639	
	Age	Stepwise	−93.042	17.138	27.864	−0.080	−3.339	<0.001	−147.713	−38.371	1.085
		Bootstrap	−110.180		30.497			0.001	−173.222	−44.991	

Dependent Variable: Total costs—transformed. Variables excluded in model 1: Age, sex, Charlson Comorbidity Index NRS-2002 Classification. Variables excluded in the model 2: Age, Sex, Charlson Comorbidity Index. Results of Linear Regression Analysis using the stepwise method and bootstrap resampling (1,000 replications, BCa method).

For costs with medicines, nutrition costs, antibacterial costs and costs with daily hospitalization, the main regressors were length of hospitalization and nutritional risk. Regarding costs with complementary diagnostic methods, the regressors were length of hospitalization (the most important) and age (Supplementary Table S2).

The cost structure analysis revealed that daily hospitalization costs were the largest contributor to total costs, followed by medication costs (Supplementary Table S3).

A significant association was detected between nutritional status and spending on nutritional support ( $\chi_{1}^{2}=71.033,p<0.001$ and effect size = 0.248), showing that when there is no risk of malnutrition, there is a tendency for there to be no spending on nutritional support. However, these results reveal an important fact: of the 488 patients at risk of malnutrition, 74.4% (*n* = 363) did not have any costs on nutritional support (Table 4).

**Table 4:** Study of the relationship between nutritional risk and spending on nutritional support.

Nutritional risk			Nutritional support costs		Total	Test statistics			Effect size
			No	Yes		Qui-square	df	*p*	
NRS-2002 classification	Without nutritional risk	*n*	612	50	662	71.033^a^	1	<0.001*	0.248^1^
		% within NRS-2002 classification	92.4%	7.6%	100%				
	With nutritional risk	*n*	363	125	488				
		% within NRS-2002 classification	74.4%	25.6%	100%				
Total		*n*	975	175	1,150				
		% within NRS-2002 classification	84,8%	15.2%	100.0%				

*Significant association at the 5% significance level. ^1^Cramer’s V. Results of the Chi-Square test.

Given that 74.4% (*n* = 363) of patients at nutritional risk and did not receive nutritional care (incurring no expenses on nutritional support), only patients at nutritional risk are considered now (*n* = 488). In an univariate analysis, no significant association was detected between mortality and whether or not there were costs for nutritional support ( $\chi_{1}^{2}=0.294,p=0.588$ and effect size = 0.020). The length of hospital stays for those with nutritional support costs was significantly longer than for those with no nutritional supplements (*U* = 11,683.5, *p* < 0.001 and effect size = 0.400). No significant association was detected between nutritional support costs and readmissions up to 30, 90, and 180 days (*p*’s > 0.05 and very small effect sizes) (Table 5).

**Table 5:** Study of the relationship between mortality, length of hospital stays and readmissions up to 30, 90, or 180 days with the costs of nutritional support.

Deaths and readmissions			Nutritional support costs		Total	Test statistics			Effect size
			No	Yes		Qui-square	df	*p*	
Died	No	*n*	255	91	346	0.294	1	0.588^a^	0.020^1^
		% of Total	52.3%	18.6%	70.9%				
	Yes	*n*	108	34	142				
		% of Total	22.1%	7.0%	29.1%				
Total		*n*	363	125	488				
		% of Total	74,4%	25.6%	100.0%				
Length of stay	*N*		363	125		11683.5		<0.001^b,^ **	0.400^2^
	Mean Rank		214.186	332.532					
Readmissions up to 30 days	No	*n*	343	113	456	2.539	1	0.111 ^a^	0.07^1^
		% of Total	70.3%	23.2%	93.4%				
	Yes	*n*	20	12	32				
		% of Total	4.1%	2.5%	6.6%				
Total		*n*	363	125	488				
		% of Total	74,4%	25.6%	100.0%				
Readmissions up to 90 days	No	*n*	333	111	444	0.977	1	0.323 ^a^	0.05^1^
		% of Total	68.2%	22.7%	91.0%				
	Yes	*n*	30	14	44				
		% of Total	3.7%	1.6%	5.3%				
Total		*n*	363	125	488				
		% of Total	74,4%	25.6%	100.0%				
Readmissions up to 180 days	No	*n*	326	110	436	0.319	1	0.572 ^a^	0.03^1^
		% of Total	66.8%	22.5%	89.3%				
	Yes	*n*	37	15	52				
		% of Total	3.5%	1.4%	4.9%				
Total		*n*	363	125	488				
		% of Total	74,4%	25.6%	100.0%				

*Significant association at the 5% significance level. **Statistically significant differences at the 1% significance level. ^a^Chi-Square Test. ^b^Mann–Whitney test. ^1^Cramer’s V. ^2^ $\frac{|z|}{\sqrt{\text{total}\;n}}.$ Results of the Chi−Square test and Mann–Whitney test.

Considering all patients again, a time-dependent Cox regression model was fitted to evaluate the association between comorbidity burden, nutritional risk, nutritional support, and in-hospital mortality. The Charlson Comorbidity Index showed a significant and time-constant effect, with each one-point increase associated with a 10.1% higher hazard of death (HR = 1.10; 95% CI: 1.06–1.15; *p* < 0.001). In contrast, both nutritional-risk categories exhibited strong non-proportional effects, requiring the inclusion of interaction terms with time. At admission (time zero), patients with nutritional risk who did not receive nutritional supplements had a markedly increased hazard of death (HR = 23.32; 95% CI: 13.09–41.56; *p* < 0.001), while those who received nutritional supplements showed a six-fold higher hazard (HR = 6.15; 95% CI: 2.96–12.80; *p* < 0.001) compared to patients without nutritional risk. The time-interaction terms were both negative and highly significant (HR per day: 0.916 for “risk without supplements” and 0.965 for “risk with supplements”; both *p* < 0.001), indicating that the excess hazard associated with nutritional risk diminished progressively throughout the hospital stay (Table 6). The time-varying hazard-ratio curves confirmed this pattern: the “risk without supplements” group displayed a steep early decline, with the HR decreasing from >20 at admission to approximately 1.0 by day 50–60. The “risk with supplements” group showed a more gradual decrease, with the HR approaching unity around day 80–100. Confidence intervals widened at longer lengths of stay, reflecting the decrease in sample size over time (Supplementary Figure S2). Overall, the extended Cox model demonstrated substantial early mortality risk among nutritionally vulnerable patients—particularly those not receiving nutritional supplements—with risk attenuating as hospitalization progressed.

**Table 6:** Study of the relationship between hospital mortality and nutritional risk (no/yes), nutritional support (no/yes) and Charlson comorbidity index.

Variables in the model	*B*	SE	Wald	df	*p*	Hazard ratio	95.0% CI for hazard ratio	
							Lower	Upper
Charlson comorbidity index	0.096	0.021	21.191	1	<0.001	1.101	1.057	1.147
Time*With nutritional risk and without nutritional supplements	−0.088	0.011	63.858	1	<0.001	0.916	0.897	0.936
Time*With nutritional risk and with nutritional supplements	−0.035	0.008	21.050	1	<0.001	0.965	0.951	0.980
Nutritional risk combined with nutritional supplements			116.033	2	<0.001			
With nutritional risk and without nutritional supplements	3.149	0.295	114.175	1	<0.001	23.321	13.088	41.557
With nutritional risk and with nutritional supplements	1.817	0.374	23.638	1	<0.001	6.153	2.958	12.799

Results of Cox Regression with Forward Conditional method for selecting variables to be included in the model.

**Figure 1:**
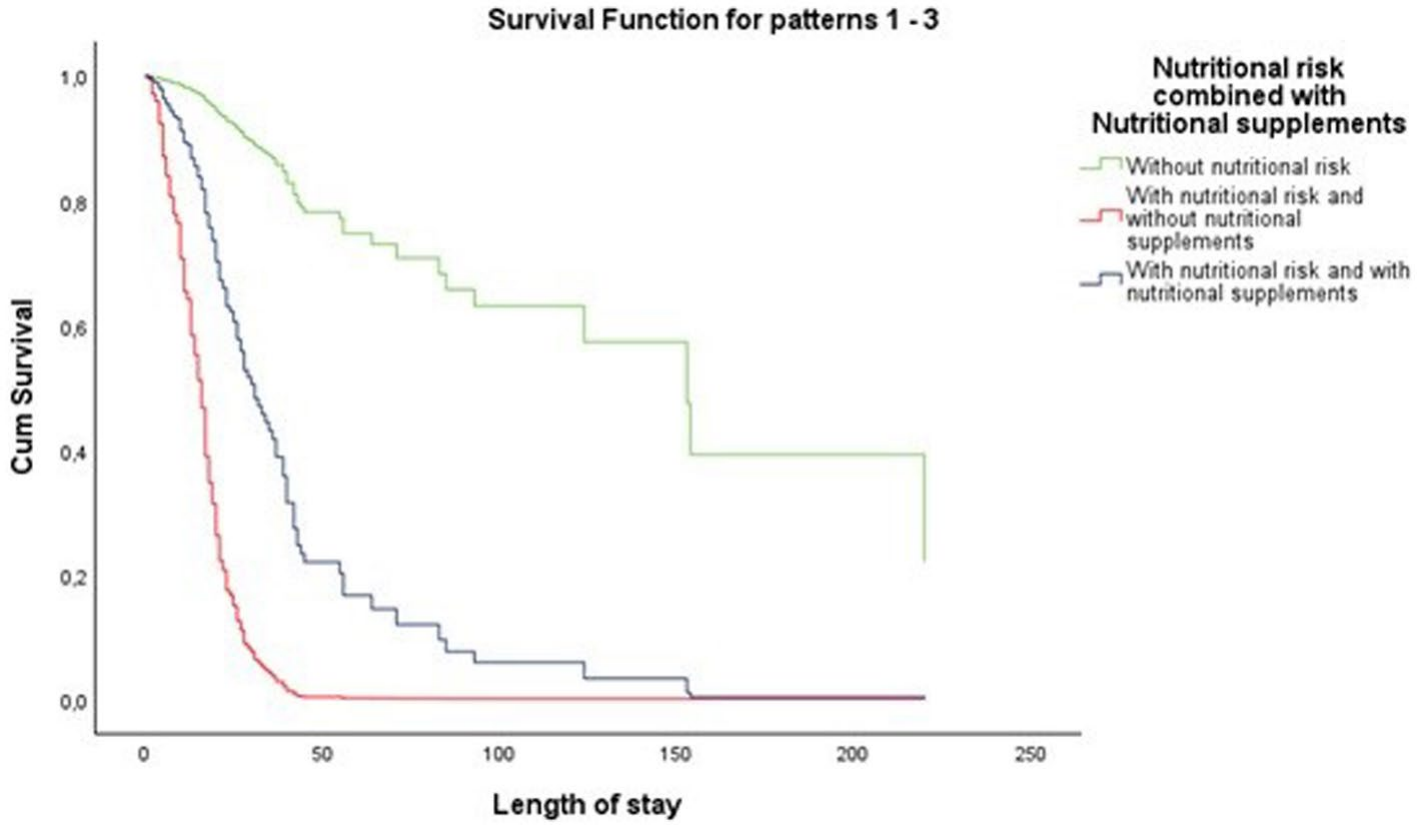
Cox regression results: hospital survival function for nutritional risk combined with nutritional supplements. Reference category: without nutritional risk.

As shown in Figure 1, patients at nutritional risk who did not receive nutritional supplementation have a lower survival rate. The survival rate of patients at nutritional risk who received nutritional supplementation is substantially higher than that of patients at nutritional risk who did not receive supplementation, but markedly lower than that of patients without nutritional risk.

After hospital discharge, a time-dependent Cox regression model was estimated to assess the association between comorbidity burden, nutritional risk, nutritional support, and mortality after hospital discharge for up to 1 year. The Charlson Comorbidity Index was significantly associated with post-discharge mortality and retained a proportional, time-constant effect (HR = 1.04; 95% CI: 1.00–1.08; *p* = 0.030), indicating that each additional comorbidity increased the hazard of death by approximately 4%. In contrast, both categories of nutritional risk violated the proportional hazards assumption, requiring the inclusion of time-interaction terms to model the changing effect of nutritional vulnerability over the follow-up period. At the time of discharge (time zero), patients classified as having nutritional risk and *not* receiving nutritional support had a markedly elevated hazard of death (HR = 10.58; 95% CI: 7.47–14.98; *p* < 0.001) compared with patients without nutritional risk. Those with nutritional risk and receiving nutritional support also exhibited a significantly increased early hazard (HR = 9.02; 95% CI: 5.73–14.21; *p* < 0.001), although slightly lower than the non-supplemented group. The time-interaction terms for both categories were statistically significant and negative, indicating a progressive attenuation of the excess hazard over time (HR per day: 0.955 for “nutritional risk without support” and 0.984 for “nutritional risk with support”; both *p* < 0.01) (Table 7). These results show that the elevated mortality risk associated with nutritional vulnerability is greatest shortly after discharge and decreases as follow-up progresses. Time-varying hazard-ratio curves with 95% confidence intervals confirmed these patterns. Individuals with nutritional risk and no nutritional support showed the steepest early decline in risk, with hazard ratios falling from >10 at discharge to approximately 1 by around 120–150 days. Those receiving nutritional support displayed a more gradual reduction in risk, with HRs declining from approximately 9 to near 1 over a broader timeframe. Confidence intervals widened at later follow-up due to decreasing numbers of events, but overall trends remained consistent (Supplementary Figure S3). These findings suggest that nutritional risk at discharge is a strong predictor of early post-discharge mortality and that the influence of nutritional status evolves substantially throughout the follow-up period.

**Table 7:** Study of the relationship between mortality after 1 year of hospital discharge and nutritional risk (no/yes) combined with nutritional support (no/yes), Charlson comorbidity index, severity of the disease, and relative weight.

Variables in the model	*B*	SE	Wald	df	*p*	Hazard ratio	95.0% CI for Hazard ratio	
							Lower	Upper
Charlson comorbidity index	0.041	0.019	4.727	1	0.030	1.042	1.004	1.081
Time*With nutritional risk and without nutritional supplements	−0.046	0.010	21.797	1	<0.001	0.955	0.936	0.973
Time*With nutritional risk and with nutritional supplements	−0.016	0.005	8.762	1	0.003	0.984	0.973	0.995
Nutritional risk combined with Nutritional supplements			186.669	2	<0.001			
With nutritional risk and without nutritional supplements	2.359	0.177	176.964	1	<0.001	10.580	7.474	14.978
With nutritional risk and with nutritional supplements	2.200	0.232	90.054	1	<0.001	9.021	5.728	14.209

Results of Cox Regression with time truncated to the left at 365 days.

**Figure 2:**
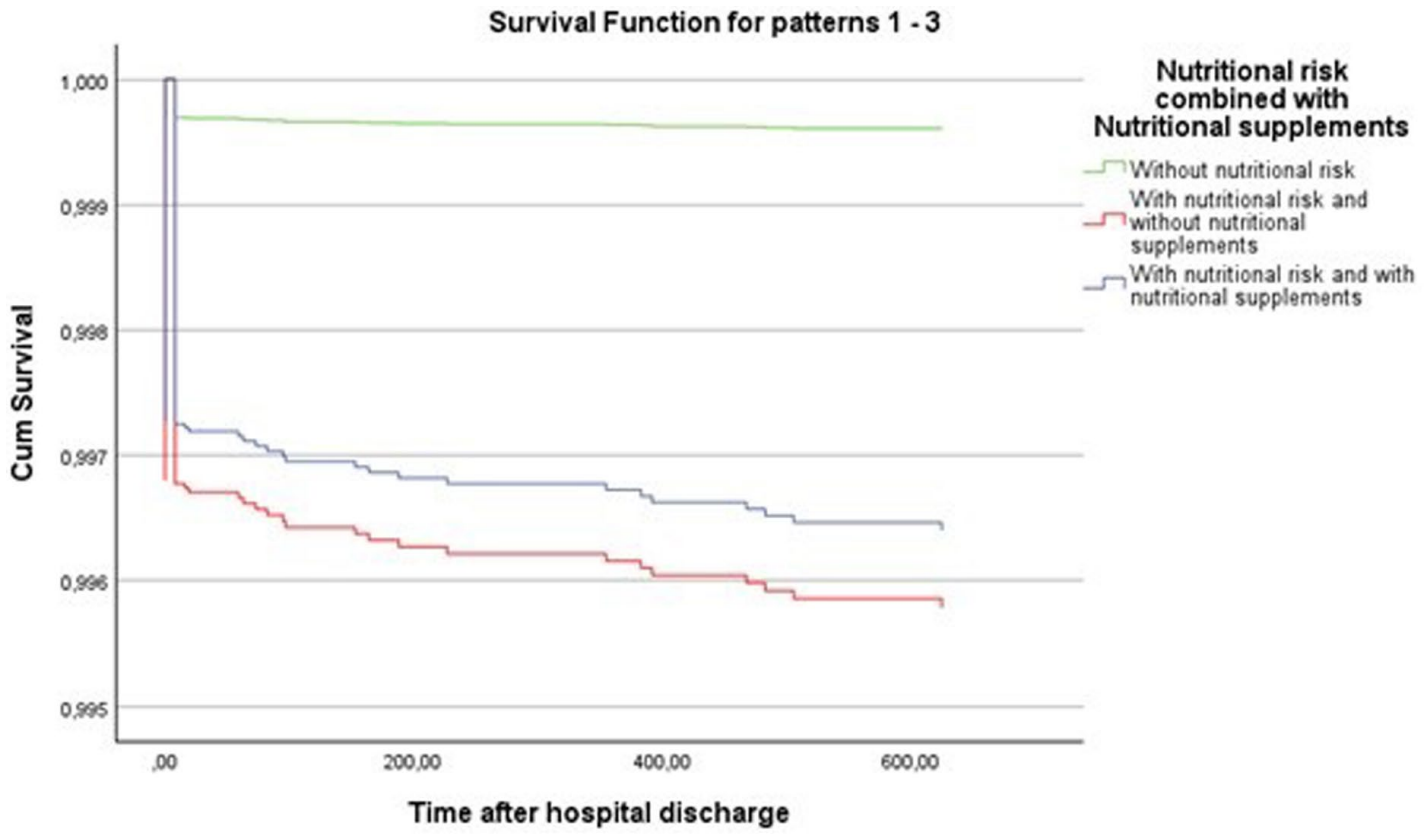
Cox regression results: survival function 1 year after hospital discharge for nutritional risk combined with nutritional support, with time truncated to the left at 365 days. Reference category: without nutritional risk.

After hospital discharge, survival among patients at nutritional risk, in particular those who did not receive nutritional support, showed a substantially lower survival rate, whereas individuals without nutritional risk exhibited the highest and most stable survival throughout follow up (Figure 2).

In patients at nutritional risk, and in relation to the length of hospital stay and number of readmissions, it cannot be stated that the fact that patients receive nutritional supplementation brings an advantage. Based on the observed hazard ratios and cohort size, a counterfactual estimate suggests that up to 9 deaths (95% CI: 3–15) among patients at nutritional risk may have been associated with the absence of nutritional supplementation; however, this estimate derives from an observational analysis and should not be interpreted as a demonstration of causal preventability (Table 8).

**Table 8:** Descriptive statistics for in-hospital mortality, length of stay and readmissions up to 30, 80, and 180 days in patients with nutritional risk, without and with nutritional support.

Nutritional risk and nutritional support	Outcomes	*n*	%	Minimum	Maximum	Mean	Standard deviation
With nutritional risk and without nutritional support (*n* = 363)	In-hospital mortality	108	29.8%				
	Length of stay			1	430	17.88	27.65
	Readmissions up to 30 days	20	5.5%				
	Readmissions up to 90 days	30	8.3%				
	Readmissions up to 180 days	37	10.2%				
With nutritional risk and with nutritional support (*n* = 125)	In-hospital mortality	34	27.2%				
	Length of stay			2	320	44.64	52.09
	Readmissions up to 30 days	12	9.6%				
	Readmissions up to 90 days	14	11.2%				
	Readmissions up to 180 days	15	12.0%				

## 4 Discussion

Our findings demonstrate a nutritional risk prevalence of 42.4% among Internal Medicine patients, which aligns closely with existing literature reporting rates between 40 and 55% in similar populations (1, 16, 17). This prevalence compares favorably with the 51% reported in the multicenter ANUMEDI study across 24 Portuguese hospitals, using the same tools (18). Of particular concern is the dramatic discrepancy between the 42.4% prevalence identified through systematic screening and the mere 0.7% ICD-10 coding rate documented in discharge summaries. This finding mirrors the observations by Marco et al. (19), who reported similarly low coding rates despite high prevalence, and aligns with validation studies showing poor agreement between diagnostic codes and standardized screening tools (20). The implementation of mandatory nutritional screening in Portugal increased awareness and coding rates from 10.6 to 25.4% over time (21), suggesting that systematic screening programs can improve recognition and documentation.

The independent prognostic value of nutritional risk observed in our study is consistent with extensive evidence showing increased mortality among malnourished or nutritionally vulnerable patients. In our cohort, individuals with nutritional risk who did not receive nutritional support had a 23.3-fold higher hazard of in-hospital death compared with patients without nutritional risk, and a 10.6-fold higher hazard of post-discharge mortality. These strong early effects, which diminish over time, are in line with previous validation studies of the NRS-2002 tool and reinforce the clinical relevance of timely nutritional assessment and intervention. A large Italian cohort study of 5,698 patients found that NRS-2002 ≥ 3 was an independent predictor of mortality across multiple time intervals, with hazard ratios consistently exceeding 1.4 (11). Similarly, the 5-year follow-up of the EFFORT trial demonstrated that nutritional risk scores showed a stepwise increase in mortality risk, with adjusted hazard ratios of 1.28 for 1-year mortality and 1.13 for long-term mortality (22).

The prognostic significance of nutritional risk extends beyond immediate hospitalization outcomes. Studies in specific populations, such as community-acquired pneumonia patients, have shown that malnutrition remains an independent predictor of 2-year mortality with odds ratios of 2.52 (23). Meta-analyses of nutritional risk indices consistently demonstrate hazard ratios ranging from 1.56 to 2.10 for all-cause mortality across different patient populations (24, 25), reinforcing the robustness of the survival signal associated with nutritional risk.

The economic analysis reveals that nutritional risk substantially increases hospital resource utilization, with costs nearly doubling for patients at risk, mainly driven by longer length of stay (LOS) and higher medication, diagnostic, and antibacterial costs. Daily hospitalization costs were the largest contributor, increasing approximately 83% in nutritionally at-risk patients. The Canadian Malnutrition Task Force study found that moderately malnourished patients had 18% longer hospital stays and 31–34% higher costs, while severely malnourished patients had 34% longer stays and 38% higher costs (26). These findings are remarkably consistent with our observation that the daily hospitalization costs were the largest contributor, increasing approximately 83% in nutritionally at-risk patients.

The economic burden of hospital malnutrition has been quantified globally, with studies reporting additional costs of US$1,500–2,000 per hospital stay in Canada (9), estimated annual costs of $30.1 billion across Asian countries (7), and £7.3 billion annually in the UK (8). The cost structure identified in our study, with daily hospitalization costs representing the largest contributor, followed by medication costs, aligns with these international analyses demonstrating that prolonged length of stay is the primary driver of excess costs associated with malnutrition. Such findings emphasize that malnutrition is not only a clinical risk factor but also a significant economic burden that warrants prioritized identification and management. The Portuguese National Health System (SNS) currently operates under a population-based financing model using the ACG (Adjusted Clinical Groups) risk stratification framework developed by Johns Hopkins University. Morbidity coding, including malnutrition, directly impacts the population risk coefficient attributed to each Local Health Unit (Unidade Local de Saúde), with financial consequences on per-capita allocation. Under-coding of malnutrition (0.7% ICD-10 coding rate in this cohort versus 42.4% NRS-2002 positivity) therefore represents not only a clinical governance failure but a systematic financial underestimation within this model. While direct extrapolation to other healthcare systems requires caution, the principle that malnutrition coding gaps translate into under-resourced care pathways is likely generalizable across DRG-based and capitation-based financing models internationally.

These data advocate for health system policies to incorporate routine nutritional screening protocols and timely individualized nutritional support, as recommended by ESPEN guidelines, to optimize patient outcomes and resource use (14). Recent health economic evaluations provide compelling evidence for the cost-effectiveness of nutritional interventions in hospitalized patients. A comprehensive cost-consequence analysis demonstrated that complex nutritional interventions targeting malnutrition in hospital settings cost approximately $219 per patient during hospitalization, with the potential to avert significant disability-adjusted life years (DALYs). When extended to 3-month post-discharge programs, additional costs of $814 per patient yielded substantial long-term benefits, including reduced readmissions and mortality (27). The EFFORT trial’s economic evaluation revealed that in-hospital nutritional support cost approximately $36 per patient across the hospital stay, while generating substantial cost savings through reduced mortality (8.3% vs. 11.0%) and complications. The analysis demonstrated that nutritional support was cost-effective for reducing readmission risk and hospital-associated complications, with total healthcare costs averaging $63,227 per patient in the intervention group, compared with higher downstream costs in untreated patient (9).

It is important to emphasize that this study did not include a formal cost-effectiveness or cost-utility analysis, and no direct demonstration of cost savings or cost neutrality was made within this dataset. The finding that nutritional supplementation was associated with increased admission costs, driven predominantly by longer length of stay in sicker patients, should not be interpreted as evidence against cost-effectiveness. The potential value of avoided deaths represents a societal gain that was not quantified in this study. Readers are directed to published cost-consequence analyses for formal economic evaluations.

The mortality patterns observed in our cohort provide compelling evidence for the clinical importance of nutritional intervention in medically ill hospitalized patients. Individuals at nutritional risk who did not receive nutritional support exhibited a 23.3-fold higher hazard of in-hospital mortality compared with patients without nutritional risk, whereas those receiving nutritional supplementation still demonstrated increased risk but to a lesser extent (HR = 6.15, 95% CI: 2.96–12.80). These findings suggest that nutritional support may attenuate—but not fully eliminate—the excess short-term mortality risk associated with nutritional vulnerability. This protective trend extended beyond hospitalization: after discharge, patients with nutritional risk who were not supplemented experienced a 10.6-fold higher hazard of post-discharge mortality, highlighting persistent vulnerability in the absence of nutritional intervention. Collectively, these results reinforce the growing body of evidence supporting systematic nutritional screening and timely supplementation as essential components of comprehensive inpatient care.

The landmark EFFORT trial demonstrated a 35% reduction in 30-day mortality among 2,028 patients randomized to individualized nutritional support (28). Similarly, the NOURISH study showed a 51% reduction in 90-day mortality among patients receiving specialized oral nutritional supplements (29). An updated meta-analysis of 27 trials including 6,803 patients confirmed that nutritional support was associated with significantly lower mortality rates (8.3% vs. 11.0%; OR 0.73, 95% CI: 0.56–0.97) and reduced nonelective hospital readmissions (14.7% vs. 18.0%; risk ratio 0.76, 95% CI: 0.60–0.96). The absolute mortality benefit of 2.8% corresponded to a number needed to treat of 36 to prevent one death. Notably, sensitivity analyses revealed more pronounced mortality reductions in recent trials published after 2015 (OR 0.47, 95% CI: 0.28–0.79) compared to older studies, and in patients with established malnutrition (OR 0.52, 95% CI: 0.34–0.80) compared to those at nutritional risk (12). A large population-based cohort study of over 110,000 patients with malnutrition demonstrated a 21% relative risk reduction in in-hospital mortality (IRR 0.79, 95% CI: 0.75–0.84) among patients receiving nutritional support, with mortality rates of 7.2% versus 8.8% in matched cohorts. These findings remained consistent across different nutritional support modalities, with oral nutritional support alone associated with a 25% reduction in overall in-hospital mortality risk (29).

However, our finding that 74.4% of patients at nutritional risk did not receive nutritional supplementation reveals a significant care gap that has been documented internationally. Studies have reported that between 30 and 80% of patients identified as at nutritional risk through screening do not receive appropriate nutritional intervention (13, 30, 31). This implementation gap may be explained by multiple barriers including lack of knowledge among healthcare providers, inadequate resources, poor interdisciplinary communication, and competing clinical priorities (32, 33). In our hospital’s case, data collection occurred during a transition phase when the nutritional screening and evaluation pathway was undergoing improvement, which may have contributed to communication gaps and suboptimal intervention rates.

This study possesses several notable strengths. First, it is based on a large, well-characterized cohort of 1,150 internal medicine inpatients, which enhances the statistical power and generalizability of the findings. The use of validated instruments such as the NRS-2002 for nutritional risk assessment and the Charlson Comorbidity Index for evaluating comorbidity burden ensures methodological rigor and comparability with existing research with comprehensive data collection allowed for robust analyses of both clinical outcomes (mortality, readmissions) and economic impacts (detailed cost breakdowns). However, the study also has important limitations. Its retrospective, single-center design limits the ability to establish causal relationships and may reduce generalizability to other healthcare settings; however, the consistency of our findings with prior controlled trials supports their clinical relevance. The requirement for NRS-2002 screening within 48 h of admission introduced a structural selection bias. Patients admitted during weekends or directly from the Emergency Department are systematically less likely to undergo timely screening. Similarly, terminally ill patients are not routinely screened. These groups are likely to have higher nutritional risk prevalence and greater illness severity. Their systematic exclusion may lead to underestimation of true nutritional risk prevalence and a “healthier-than-average” analytic cohort relative to the broader hospital population. Another important limitation concerns the definition of nutritional supplementation, in this retrospective dataset, nutritional support could only be inferred indirectly from admission-level expenditure on nutritional products recorded in the pharmacy and costing systems, which aggregate oral supplements, enteral tube feeds and parenteral nutrition. As a result, we were unable to distinguish between different routes, doses or duration of medical nutrition therapy, and we could not capture non-product-based interventions such as individualized dietetic counselling. This exposure misclassification may attenuate or distort the observed associations between supplementation and clinical outcomes and prevents firm conclusions regarding the comparative effectiveness of specific nutrition modalities. Unmeasured confounding represents a major limitation. Clinically important variables such as functional status, frailty, inflammatory biomarkers (e.g., CRP, albumin), acute illness severity scores (e.g., NEWS), and socioeconomic factors were not captured in the administrative dataset. A critical concern is confounding by indication: patients who received nutritional supplementation were likely clinically distinct from those who did not, possibly with longer hospital stays favoring intervention opportunities, under closer clinical surveillance, or with differing baseline prognosis. The direction and magnitude of this bias are difficult to quantify without individual-level clinical complexity data, but it is plausible that the observed protective association of supplementation is partially attenuated by residual confounding. Additionally, survivorship bias cannot be excluded: patients who died early in admission had less opportunity to receive nutritional support, potentially inflating the apparent protective effect. Propensity score matching or instrumental variable analyses in future prospective studies would help address these limitations. Finally, the observed care gap between identification and intervention may be influenced by local practice patterns, resource availability, and documentation practices, which may differ in other institutions. These limitations should be considered when interpreting the study’s findings and their implications for clinical practice.

Future research should focus on implementation science approaches to improve the translation of screening results into effective interventions. Multidisciplinary education, combined with systematic integration of nutritional risk screening tools into electronic health records and care pathways, may improve intervention rates and outcomes (32). Additionally, extending nutritional support beyond discharge, such as outpatient supplementation and meal-delivery programs, holds promise to further reduce long-term mortality and hospital readmissions, albeit requiring additional evidence to guide best practice (12). Randomized controlled trials specifically designed to test different implementation strategies for nutritional care pathways are needed. The development and validation of predictive models that can identify patients most likely to benefit from nutritional intervention could help optimize resource allocation and improve cost-effectiveness.

## 5 Conclusion

This study confirms that nutritional risk affects 42.4% of Internal Medicine patients and independently predicts mortality, with unsupplemented at-risk patients facing a 23.3-fold increased risk of death. The critical finding that 74.4% of at-risk patients received no nutritional intervention represents a substantial missed opportunity that could have prevented an estimated 9 deaths in our cohort. While nutritional supplementation increased hospital costs by 79% patients who received supplementation showed a pattern of lower mortality risk than unsupplemented at‑risk patients, although mortality remained higher than in patients without nutritional risk. These findings are consistent with, but do not prove, a beneficial association of nutritional support. While a formal cost-effectiveness analysis was not conducted in this study, the observed association between nutritional risk and increased resource utilization, combined with the potential mortality benefit of supplementation, suggests systemic value from a societal perspective that warrants further economic evaluation.

## Data Availability

The raw data supporting the conclusions of this article will be made available by the authors, without undue reservation.

## Supplementary material

Supplementary Figure S1 Flowchart of the patient sampling.

Supplementary Figure S2 Time‑varying hazard ratios [HR(t)] during length stay.

Supplementary Figure S3 Time-varying hazard ratios [HR(t)] after hospital discharge.

Supplementary TABLE S1 Mortality after hospital discharge, up to one year of follow-up.

Supplementary TABLE S2 Identification of predictors of various types of costs. Results of Linear Regression Analysis using the stepwise method.

Supplementary TABLE S3 Identification of total cost predictors among the different types of costs. Results of Linear Regression Analysis.
