# Supplementary figures and images for: Missed opportunities in nutritional care: prevalence, mortality, and resource utilization in internal medicine wards
Source: Front Nutr. 2026 May 13;13:1755750. doi: 10.3389/fnut.2026.1755750 (PMC13212179; doi:10.3389/fnut.2026.1755750)

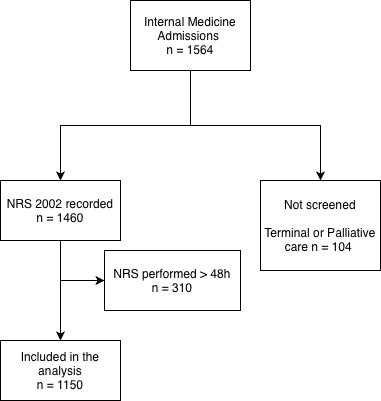

Supplement: Supplementary Figure S1 — Flowchart of the patient sampling. [file Image_1.jpeg]

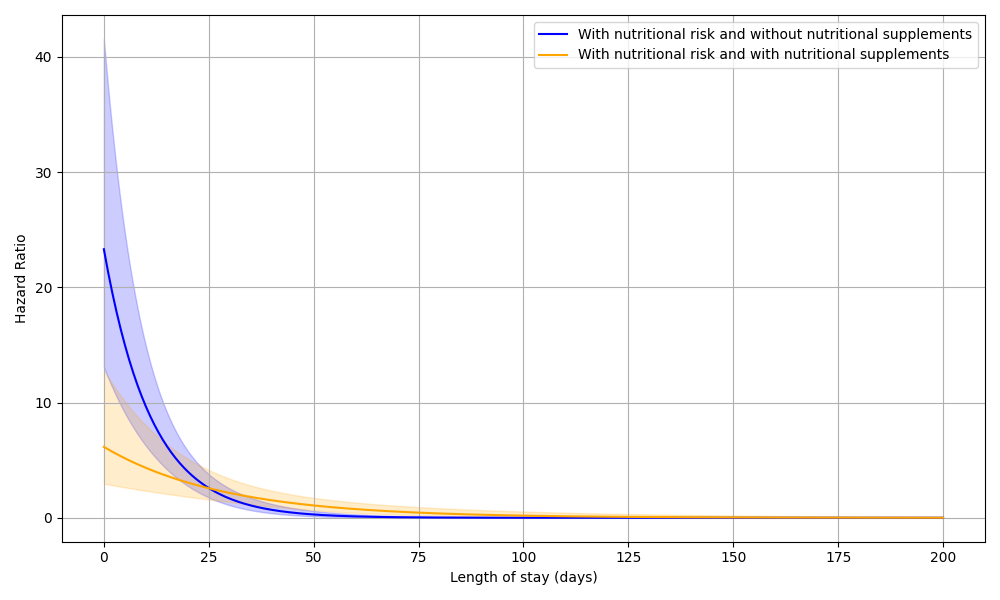

Supplement: Supplementary Figure S2 — Time‑varying hazard ratios [HR(t)] during length stay. [file Image_2.png]

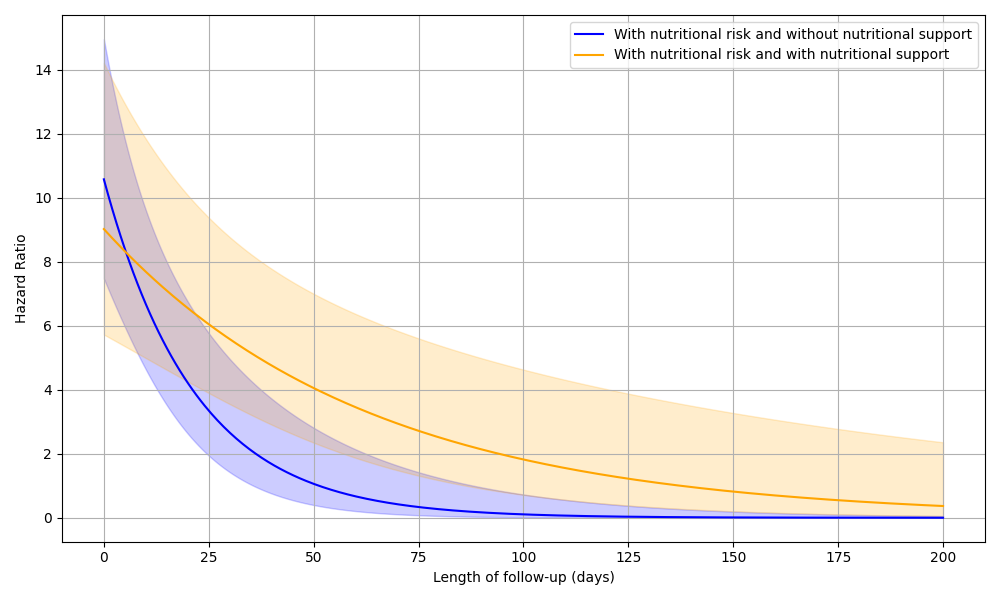

Supplement: Supplementary Figure S3 — Time-varying hazard ratios [HR(t)] after hospital discharge. [file Image_3.png]
