# Supplementary material for: Missed opportunities in nutritional care: prevalence, mortality, and resource utilization in internal medicine wards
Source: Front Nutr. 2026 May 13;13:1755750. doi: 10.3389/fnut.2026.1755750 (PMC13212179; doi:10.3389/fnut.2026.1755750)
Supplement: Supplementary TABLE S1 — Mortality after hospital discharge, up to one year of follow-up. [file Table_1.docx]

Table S1: Mortality after hospital discharge, up to one year of follow-up

|  | n | Number of deaths | % |
| --- | --- | --- | --- |
| Death up to 30 days after discharge | 1150 | 69 | 6,0 |
| Death between 30 and 60 days after discharge | 894 | 95 | 10,6 |
| Death between 60 and 180 days after discharge | 829 | 56 | 6,8 |
| Death between 180 and 365 days after discharge | 733 | 65 | 8,9 |
| Death after 1 year | 663 | 131 | 19,8 |
