# Supplementary material for: Missed opportunities in nutritional care: prevalence, mortality, and resource utilization in internal medicine wards
Source: Front Nutr. 2026 May 13;13:1755750. doi: 10.3389/fnut.2026.1755750 (PMC13212179; doi:10.3389/fnut.2026.1755750)
Supplement: Supplementary TABLE S2 — Identification of predictors of various types of costs. Results of Linear Regression Analysis using the stepwise method. [file Table_2.docx]

Table S2: Identification of predictors of various types of costs. Results of Linear Regression Analysis using the stepwise method.

| Dependent variable | Model | Variables in equation | Unstandardized Coefficients | | Standardized Coefficients | t | p | 95,0% Confidence Interval for B | | Collinearity Statistics |
| --- | --- | --- | --- | --- | --- | --- | --- | --- | --- | --- |
|  |  |  | B | Std. Error | Beta |  |  | Lower Bound | Upper Bound | VIF |
| Medication costs - transformed | 4 | (Constant) | 2532.615 | 797.288 |  | 3.177 | 0.002 | 968.304 | 4096.926 |  |
|  |  | Length of stay | 79.805 | 6.027 | 0.361 | 13.241 | 0.000 | 67.980 | 91.630 | 1.028 |
|  |  | NRS2002 Classification | 1869.668 | 394.300 | 0.135 | 4.742 | 0.000 | 1096.036 | 2643.300 | 1.122 |
|  |  | Age | -59.997 | 12.137 | -0.151 | -4.943 | 0.000 | -83.811 | -36.182 | 1.298 |
|  |  | Charlson Comorbidity Index | 207.206 | 61.897 | 0.100 | 3.348 | 0.001 | 85.762 | 328.650 | 1.244 |
|  | Variables excluded in the model 1: Age, sex, Charlson comorbidity index, NRS2002 classification. Variables excluded in the model 2:Age, sex, Charlson comorbidity index. Variables excluded in the model 3: Age, sex. | | | | | | | | | |
| Nutrition costs - transformed | 4 | (Constant) | 9.874 | 4.043 |  | 2.442 | 0.015 | 1.940 | 17.807 |  |
|  |  | Length of stay | 0.682 | 0.056 | 0.329 | 12.131 | <0.001 | 0.572 | 0.793 | 1.028 |
|  |  | NRS2002 Classification | 27.617 | 3.603 | 0.212 | 7.665 | <0.001 | 20.548 | 34.687 | 1.069 |
|  |  | Sex | -10.250 | 3.482 | -0.079 | -2.944 | 0.003 | -17.082 | -3.418 | 1.002 |
|  |  | Charlson Comorbidity Index | -1.289 | 0.530 | -0.066 | -2.431 | 0.015 | -2.329 | -0.249 | 1.040 |
|  | Variables excluded in the model 1: Age, sex, Charlson comorbidity index, NRS2002 classification. Variables excluded in the model 2:Age, sex, Charlson comorbidity index. Variables excluded in the model 3: Age, Charlson comorbidity index. Variables excluded in the model 4: :Age | | | | | | | | | |
| Antibacterial costs - transformed | 3 | (Constant) | 60.594 | 52.527 |  | 1.154 | 0.249 | -42.466 | 163.654 |  |
|  |  | Length of stay | 6.330 | 0.935 | 0.195 | 6.767 | <0.001 | 4.495 | 8.165 | 1.026 |
|  |  | NRS2002 Classification | 305.919 | 58.624 | 0.150 | 5.218 | <0.001 | 190.896 | 420.942 | 1.027 |
|  |  | Sex | -128.182 | 57.797 | -0.063 | -2.218 | 0.027 | -241.583 | -14.782 | 1.001 |
|  | Variables excluded in the model 1: Age, sex, Charlson comorbidity index, NRS2002 classification. Variables excluded in the model 2:Age, sex, Charlson comorbidity index. Variables excluded in the model 3: Age, Charlson comorbidity index. | | | | | | | | | |
| Costs for complementary diagnostic means - transformed | 2 | (Constant) | 1185.705 | 128.554 |  | 9.223 | <0.001 | 933.478 | 1437.933 |  |
|  |  | Length of stay | 13.962 | 0.979 | 0.385 | 14.255 | <0.001 | 12.040 | 15.884 | 1.001 |
|  |  | Age | -9.355 | 1.728 | -0.146 | -5.413 | <0.001 | -12.746 | -5.964 | 1.001 |
|  | Variables excluded in the model 1: Age, sex, Charlson comorbidity index, NRS2002 classification. Variables excluded in the model 2:Sex, Charlson comorbidity index, NRS2002 classification. | | | | | | | | | |
| Daily hospitalization costs - transformed | 2 | (Constant) | 404.949 | 510.489 |  | 0.793 | 0.428 | -596.648 | 1406.546 |  |
|  |  | Length of stay | 330.109 | 12.076 | 0.622 | 27.336 | <0,001 | 306.416 | 353.802 | 1.025 |
|  |  | NRS2002 Classification | 3615.586 | 745.168 | 0.110 | 4.852 | <0,001 | 2153.540 | 5077.633 | 1.025 |
|  | Variables excluded in the model 1: Age, sex, Charlson comorbidity index, NRS2002 classification. Variables excluded in the model 2: Age, Sex, Charlson comorbidity index. | | | | | | | | | |

Sensitivity analysis was also performed for these models using bootstrap resampling (1000 replications, BCa method) and alternative variable selection procedures (forward and backward stepwise). Bootstrap estimates and confidence intervals were similar to the original model, indicating robustness of the findings.
