# Supplementary material for: Missed opportunities in nutritional care: prevalence, mortality, and resource utilization in internal medicine wards
Source: Front Nutr. 2026 May 13;13:1755750. doi: 10.3389/fnut.2026.1755750 (PMC13212179; doi:10.3389/fnut.2026.1755750)
Supplement: Supplementary TABLE S3 — Identification of total cost predictors among the different types of costs. Results of Linear Regression Analysis. [file Table_3.docx]

Table S3: Identification of total cost predictors among the different types of costs. . Results of Linear Regression Analysis

| Model | Unstandardized Coefficients | | Standardized Coefficients | t | p | 95,0% Confidence Interval for B | | Collinearity Statistics |
| --- | --- | --- | --- | --- | --- | --- | --- | --- |
|  | B | Std. Error | Beta |  |  | Lower Bound | Upper Bound | VIF |
| (Constant) | 93,933 | 109,113 |  | 0,861 | 0,389 | -120,152 | 308,017 |  |
| Daily hospitalization costs - transformed | 0,947 | 0,008 | 0,766 | 112,909 | <0,001 | 0,931 | 0,964 | 2,347 |
| Medication costs - transformed | 0,657 | 0,017 | 0,224 | 38,885 | <0,001 | 0,624 | 0,690 | 1,700 |
| Costs for complementary diagnostic means - transformed | 2,414 | 0,108 | 0,134 | 22,329 | <0,001 | 2,202 | 2,626 | 1,827 |
| Antibacterial costs - transformed | -0,772 | 0,100 | -0,039 | -7,681 | <0,001 | -0,969 | -0,575 | 1,294 |
| Nutrition costs - transformed | -4,055 | 1,518 | -0,013 | -2,671 | <0,001 | -7,033 | -1,076 | 1,205 |
| Dependent Variable: Total costs - transformed | | | | | | | | |
